# Supplementary material for: Bixin Combined with Metformin Ameliorates Insulin Resistance and Antioxidant Defenses in Obese Mice
Source: Pharmaceuticals (Basel). 2024 Sep 12;17(9):1202. doi: 10.3390/ph17091202 (PMC11434661; doi:10.3390/ph17091202)
Supplement: Supplementary file 1 [file pharmaceuticals-17-01202-s001.zip › pharmaceuticals-3194630-supplementary.pdf]

Supplementary Material

Table S1. Compositions of control diet (C) and high-fat diet (HFD).

**Ingredients**	**Control diet (C) (g/100 g)**	**High-fat diet (HFD) (g/100 g)**
Starch	42.75	14.95
Casein	20	20
Dextrin	13.2	10
Sucrose	10	10
Soy oil	4	4
Lard	--	31
Cellulose	5	5
Mix of minerals (1)	3.5	3.5
Mix of vitamins (2)	1	1
L-cystine	0.3	0.30
Choline bitartrate	0.25	0.25
Total	100	100
**Energy (kcal/100 g)**	**385**	**540**

(1) Mix of minerals: calcium, phosphorus, potassium, magnesium, iron, manganese, selenium, zinc, chromium, nickel, lithium, sulfur, copper, iodine, molybdenum, silicon, chloride, fluoride, boron, and vanadium.
(2) Mix of vitamins: nicotinic acid, calcium pantothenate, pyridoxine, thiamine, riboflavin, folic acid, vitamin K, D-biotin, vitamin B12, vitamin A, vitamin D3, vitamin E, and choline.
